# Effects of Resistance Training Combined with Vitamin D Supplementation on Health-Related Variables in the Elderly: Muscle Strength, Body Composition, and Inflammatory Status

**DOI:** 10.3390/ijerph22111695

**Published:** 2025-11-10

**Authors:** Lorena Cristina Ribeiro da Rosa, Paulo de Tarso Veras Farinatti, Maria Izabel Ferreira Batista, Hilene Ribeiro Santiago Navarro Machado, Vitor Hugo Silva de Jesus, Weslen do Nascimento Dantas, Patrícia Maria Lourenço Dutra, Nádia Souza Lima da Silva

**Affiliations:** Department of Individual Sports, Institute of Physical Education and Sports, Postraduate Program in Exercise and Sport Science, Maracanã Campus, State University of Rio de Janeiro., Rio de Janeiro 20550-013, Brazil; lorena.rosa@hotmail.com (L.C.R.d.R.); ptvf1964@gmail.com (P.d.T.V.F.); batista.maria@posgraduacao.uerj.br (M.I.F.B.); jesus.vitor@graduacao.uerj.br (V.H.S.d.J.); weslen.dantaslp@hotmail.com (W.d.N.D.); pmldutra@gmail.com (P.M.L.D.); nadiaslimas@gmail.com (N.S.L.d.S.)

**Keywords:** aging, health promotion, strength training, older adults, body composition, Inflammatory markers, IL-6, TNF-α

## Abstract

Aging is associated with changes in body composition that lead to low-grade chronic inflammation, compromising the health of the elderly. This condition can be mitigated by resistance training (RT) and vitamin D supplementation, promoting the health of this population. This study aimed to investigate the effects of 12 weeks of RT combined with vitamin D supplementation on body composition, muscle strength, and inflammatory status in older adults. A total of 26 participants were randomly assigned to an Experimental Group (EG: n = 12; 11 Female; 70.6 ± 4.7 years; RT + 2000 IU/day of vitamin D) and a Control Group (EG: n = 14; 11 Female; 69.6 ± 4.6 years; RT + placebo). Both groups performed the same RT program (8 exercises; 2 sets; 10 RM, twice per week). Before and after the intervention, participants were assessed using DEXA, strength tests (sit-to-stand test and handgrip strength), and serum biomarkers (IL-6, TNF-α, 25(OH)D). Both groups showed significant strength gains, particularly in the lower limbs (*p* < 0.05 for all tests and groups), with no between-group differences in body composition or inflammatory markers (*p* > 0.05 for all tests and groups). A time × group interaction was observed for IL-6 (*p* = 0.03), with a slight reduction in the EG and an increase in the CG, although post-intervention differences were not statistically significant (*p* = 0.49). No statistically significant between-group difference in 25(OH)D change (*p* = 0.11). These findings suggest that vitamin D supplementation at the tested dose did not enhance adaptations to resistance training in older adults with normal baseline vitamin D levels. Further studies are warranted to explore potential benefits in deficient populations and with alternative dosing strategies.

## 1. Introduction

Aging, among its various accompanying changes, alters body composition by reducing muscle mass while increasing fat mass and connective tissue, a phenomenon known as sarcopenia [1], which progressively leads to diminished muscle strength and, consequently, reduced functional capacity [2].

Notably, during this process, there is also a decline in sex hormones, which, combined with the marked loss of muscle mass, can trigger the production of cytokines, particularly interleukin-6 (IL-6) and tumor necrosis factor-alpha (TNF-α), reflecting the presence of metabolic inflammation or the “meta-inflammation”, state of low-grade chronic inflammation, typically associated with metabolic disorders such as obesity, type 2 diabetes, and metabolic syndrome [3]. Contributing to this inflammatory state, aging promotes visceral fat accumulation, an endocrine-active tissue that itself secretes cytokines and other pro-inflammatory agents [4].

At the same time, aging impairs the body’s ability to synthesize vitamin D due to the thinning of the dermis and epidermis, where 7-dehydrocholesterol (7-DHC), the precursor of its synthesis, is located. In addition, the liver and kidneys also show a reduced capacity to convert 7-DHC into vitamin D [5].

Vitamin D, acting in concert with parathyroid hormone (PTH), regulates ionized calcium concentration in the blood, which, in turn, influences neuromusculoskeletal activities. Moreover, skeletal muscle fibers express vitamin D receptors, and deficiency of this compound can cause muscle weakness or even myopathy via oxidative stress in the intracellular environment—impacting mitochondrial function and oxygen consumption [5,6].

Although these phenomena are expected in advanced age, physically active older adults tend to exhibit better control of adipose tissue gain and smaller changes in fatfree mass [7,8]. This may be linked to increased energy expenditure from exercise or a higher basal metabolic rate owing to the preservation of appendicular lean mass (ALM)—that is, lean tissue in arms and legs [9].

Indeed, Grgic et al. [10] demonstrated, through a meta-analysis, that different types of resistance training induce a significant increase in muscle mass in older individuals. This hypertrophy is driven by the synthesis of new myofibrillar proteins and activation of satellite cells [11], processes that not only promote muscle growth but also enhance muscle strength, ultimately improving autonomy and quality of life in this population [10].

Similarly, vitamin D supplementation may be an effective strategy to enhance muscle strength, given vitamin D’s role in muscle regulation, its local presence in muscle tissue, and its involvement in satellite-cell differentiation and the formation of new contractile filaments [6,12]. Thus, it is plausible to hypothesize that combining resistance training with vitamin D supplementation would yield synergistic benefits. However, a meta-analysis of studies that employed both interventions in older adults reported contradictory results among subjects with normal vitamin D levels, showing a clear benefit of supplementation only in individuals with vitamin D deficiency [13]. Despite these findings, it should be noted that this meta-analysis was conducted with only seven studies, of which only one presented a low risk of bias, indicating the need for more research in order to draw more robust conclusions about vitamin D supplementation for musculoskeletal health in older adults [13], which justified the present study.

Given the ongoing controversy in recent research and the practical implications for the health and quality of life of older adults, the present study, starting from the hypothesis that vitamin D supplementation would enhance the responses induced by resistance training, aimed to investigate the effects of resistance training combined with vitamin D supplementation on body composition, muscle strength, and inflammatory status in this population.

Finding answers to the raised question becomes relevant to support public health policies aimed at promoting active and healthy aging, through the provision of supervised physical activity programs and nutritional supplementation. Initiatives that integrate resistance training and monitoring of nutritional status, including vitamin D levels, can significantly contribute to the prevention of sarcopenia and the improvement of quality of life in the elderly population, reducing the burden on health systems and promoting greater functional autonomy.

## 2. Materials and Methods

### 2.1. Study Design

This study was a randomized, double-blind (vitamin D vs. placebo) and controlled, conducted to investigate the effect of resistance training combined with vitamin D supplementation on muscle strength, muscle mass, adipose tissue, and inflammatory status in older adults. The study was registered in the Brazilian Clinical Trials Registry (ReBEC), RBR-5x7p5zy, UTN: U1111-1294-4042 and conducted and reported in accordance with the CONSORT 2025 checklist and guidelines (Appendix A).

### 2.2. Location and Ethical Approval

The research was conducted between January and December 2022 at the Physical Activity and Health Promotion Laboratory (LABSAU) of the State University of Rio de Janeiro (UERJ), with laboratory samples collected at the Pedro Ernesto University Hospital (HUPE). The study was approved by the HUPE–UERJ Ethics Committee (Opinion No. 3.655.940).

### 2.3. Sample Size Calculation

The sample size was estimated using G*Power, version 3.1.9.7 (University of Düsseldorf), based on a repeated measures ANOVA (Effect size f = 0.48; α error probability = 0.05; Power/1 − β error probability = 0.90). The minimum required sample size was 38 participants. The study began with 40 volunteers, but for various reasons explained in Section 3.1, the minimum required number was not reached by the end of the experiment.

### 2.4. Inclusion and Exclusion Criteria

The study included male and female participants aged 60 or older, with medical clearance to engage in physical exercise and vitamin D supplementation. Participants had not been engaged in resistance training for at least three months and had no prior use of vitamin D supplements. They were also free from severe motor or cognitive impairments. Exclusion criteria included: (a) non-adherence to the protocol (exercise and supplementation); (b) failure to complete all scheduled assessments.

### 2.5. Randomization and Blinding

Participants were allocated into the two study groups using stratified randomization, aiming to ensure balance between groups in terms of sex (male and female) and age group. Participants were first categorized based on these variables and then randomized within each stratum using a computer-generated random sequence. The entire randomization process was carried out by an independent researcher who was not involved in administering the interventions or conducting any study assessments, thus ensuring allocation concealment and minimizing the risk of bias. After allocation, both the intervention staff and data collectors were kept blinded to group assignment (vitamin D vs. placebo) in order to minimize observation bias. Participants were also unaware of which supplement they were receiving, as the bottles were identical and labeled only with codes generated by the compounding pharmacy responsible for preparing the supplements. This ensured a double-blind design. The procedure guaranteed proper randomization and balanced baseline characteristics between the groups.

### 2.6. Assessments

The assessments were conducted on two consecutive days, before and after the intervention. On the first day, fasting blood samples were collected in the morning (after an 8- to 12 h fasting period) by a nursing technician, with participants seated. The samples were used to determine serum concentrations of 25-hydroxyvitamin D [25(OH)D] using the ELISA method [14], as well as the inflammatory markers TNF-α and IL-6, analyzed with the Magnetic Bead Cytokine/Chemokine Panel kit (EMD Millipore) [15]. Peripheral blood from participants was collected in tubes without anticoagulant, containing a clot activator (insert tube brand here), before and after treatment. After collection, the tubes were left undisturbed for at least 30 min to allow complete clot formation. The samples were then centrifuged at 815 g for 10 min at 25 °C. The obtained serum was carefully aliquoted in triplicate and stored in an ultrafreezer at −80 °C until analysis. Each sample was thawed only once, exclusively for the vitamin D and cytokine quantification assays. Samples showing signs of hemolysis were excluded from the analyses. Vitamin D concentrations were determined in serum samples from participants, before and after treatment, using a commercial ELISA kit (Elabscience, Houston, TX, USA, Catalog No. E-EL-0012), following the manufacturer’s instructions. The detection range was 6.25–400 ng/mL. Calibration curves were generated from the supplied standards, and sample concentrations were calculated using the SkanIt for Microplate Readers RE software (ver. 6.0.2.3), with curve fitting by a four-parameter logistic (4PL) model. Absorbance was measured on a microplate reader at 450 nm. Cytokine concentrations in serum samples from participants, before and after treatment, were determined using a bead-based fluorescent cytometric assay (CBA), with the CBA Th1/Th2/Th17 Kit (BD Biosciences, San Jose, CA, USA) for the detection of IL-17A, IFN-γ, TNF, IL-10, IL-6, IL-4, and IL-2. The assay was performed according to the manufacturer’s instructions, and data acquisition was carried out using a BD (FACS) FACSCanto II flow cytometer. The standard curves for cytokines ranged from 0 to 5000 pg/mL, and the results were analyzed using FCAP Array software v3, being expressed in pg/mL.

On the second day, body mass and height were measured to calculate Body Mass Index (BMI) (mass-kg/height-m^2^), following the protocols of Martin et al. [16] and Gordon et al. [17], respectively. Body composition was assessed using Dual-Energy X-ray Absorptiometry (DEXA) (Lunar Prodigy Advance^®^, GE Healthcare, Madison, WI, USA), and the procedures followed the recommendations of the International Society for Clinical Densitometry [18]. To ensure quality control of the results, a single trained and experienced technician performed equipment calibration before the assessments, using a phantom provided by the manufacturer. Calibration results were recorded daily and monitored over time to detect potential systematic drifts of the device, which also underwent periodic technical inspections.

Lower limb strength was assessed using the 30 s chair stand test. Participants were seated in the middle of a sturdy, armless chair approximately 43 cm in height, with their feet flat on the floor and spaced shoulder-width apart. Arms were crossed over the chest, with the hands resting on the opposite shoulders, and the trunk was kept upright without touching the backrest. After the test was explained and demonstrated, participants were instructed to stand up fully (achieving full extension of the knees and hips) and sit down again as many times as possible within 30 s. If the participant was in the process of standing when the time ended, that repetition was counted. The total number of complete stands performed in 30 s was recorded as the final score [19].

Handgrip strength was assessed using a hydraulic dynamometer (Takei 5001 Hand Grip Analogue Dynamometer-Grip-A, Niigata, Japan). Participants were seated on an armless chair with the arm positioned alongside the trunk, the elbow flexed at 90°, the forearm in a neutral position, the feet flat on the floor, and the back supported by the chair’s backrest, according to the Clinical Assessment Recommendations [20]. After the test procedure was explained and demonstrated, participants were instructed to squeeze the dynamometer with their dominant hand as forcefully as possible for approximately 3 to 5 s. Three trials were performed with 15 to 30 s rest intervals between them, and the highest value was recorded for analysis.

### 2.7. Training Protocol

To determine individual resistance training loads, participants underwent 10-repetition maximum (10 RM) tests for the exercises that would be performed throughout the experiment. These tests were conducted in two sessions separated by a 48 h interval to ensure muscle recovery and participant safety. This was followed by a two-week familiarization period using submaximal loads [21].

The intervention protocol was supervised by two of the study’s authors (L.C.R.R. and M.I.F.B.), both physical education professors with master’s degrees, and by three undergraduate interns from the Physical Education program. Each intern was responsible for supervising, on average, two participants during the morning sessions.

The resistance training program lasted 12 weeks, with two sessions per week, and included eight exercises targeting the major muscle groups: leg press, bench press, leg extension, seated row, leg curl, lat pulldown, calf raise, and abdominal exercises.

Each exercise was performed in two sets of 10 repetition maximums (10 RM) with a load that prevented the execution of an eleventh repetition but did not induce concentric failure, given the elderly participants. A two-minute rest interval was allowed between sets. Whenever the load became light enough to allow up to 12 repetitions, adjustments were made to ensure a consistent training intensity throughout the intervention.

### 2.8. Nutritional Intervention

The experimental group (EG) received 2000 IU/day of vitamin D_3_ for 12 weeks, while the control group (CG) received an identical-appearing placebo (microcrystalline cellulose). Both compounds were supplied by Quintessence Homeopathy LTDA, Rio de Janeiro, Brazil (CNPJ 31.125.586/0001-44).

### 2.9. Definition of Adherence by Protocol

To monitor protocol adherence, attendance at each training session was recorded in a class diary. Additionally, daily messages were sent via a WhatsApp group asking participants whether they had taken their supplements that morning, which also served as a reminder. Responses were documented in individual records. At the end of each month, unused tablets were returned to the training supervisors. The adherence criteria were as follows: (a) failure to complete at least 70% of the training sessions (≥19 sessions); (b) failure to consume at least 80% of the prescribed vitamin D supplement doses; (c) failure to complete all scheduled assessments.

### 2.10. Outcomes

The primary outcomes were: (1) body composition (lean mass, total fat mass, and body fat percentage); (2) muscle strength (sit-to-stand test, handgrip strength, and progression of training load); and (3) inflammatory markers (IL-6 and TNF-α). No secondary outcomes were assessed.

### 2.11. Statistical Analysis

Data were initially processed through descriptive analyses of central tendency and dispersion measures. Normality was assessed using the Shapiro–Wilk test, and sphericity using Mauchly’s test. Initially, all participants were included in the analysis, even those who did not complete all assessment stages or the intervention period. A linear mixed model with missing data handling was applied, followed by Bonferroni Post Hoc tests to identify statistically significant differences. Subsequently, a per-protocol analysis was conducted, including only participants who completed all stages of the study. This sensitivity analysis was performed using a two-factor repeated-measures ANOVA, also followed by Bonferroni Post Hoc tests, to examine pre- and post-intervention differences, as well as intra- and inter-group variations. To investigate the influence of inflammatory status on post-intervention changes in functional capacity and muscle strength variables, a MANCOVA was performed, using baseline IL-6 and TNF-α levels as covariates. The percentage change (Δ%) was calculated and reported for all variables, along with mean differences and 95% confidence intervals (95% CI). A significance level of *p* ≤ 0.05 was adopted. All analyses were conducted according to protocol using SPSS software, version 25 (IBM^®^).

## 3. Results

### 3.1. Participant Flow

**Figure 1:**
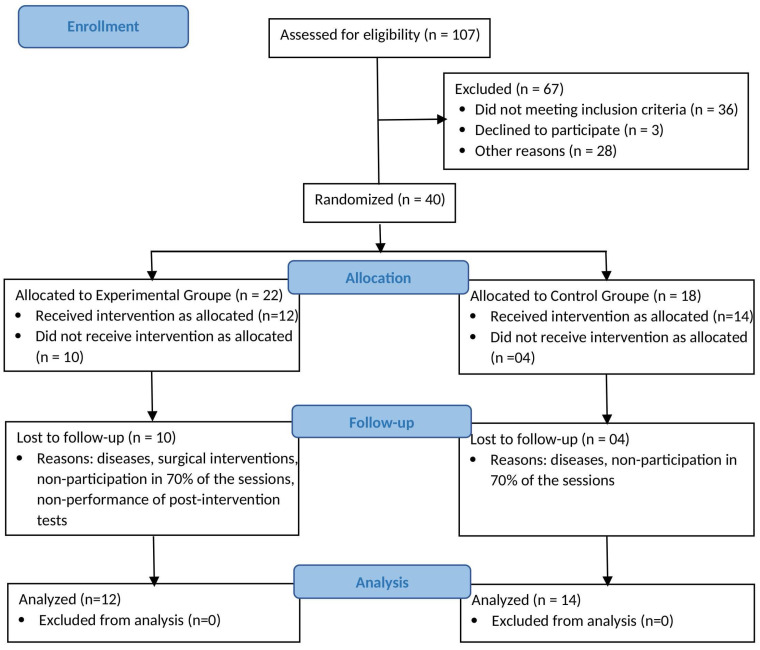
Sample Flow Diagram.

This study effectively began with the participation of 40 older adults; however, logistical challenges during the pandemic led to participant loss. Fourteen participants were excluded for reasons including illness, failure to attend at least 70% of intervention sessions, undergoing surgical procedures, and not completing post-intervention testing. The final sample comprised 26 individuals (EG: 12; CG: 14), as illustrated in the participant flow diagram (Figure 1).

### 3.2. Final Analysis

Both an intention-to-treat analysis, including all participants initially enrolled in the study (n = 40), and a per-protocol analysis, comprising only those who completed all stages of the research (n = 26), were performed. As both analyses yielded comparable results, and the baseline differences observed among participants who completed the study—specifically in TNF-α and IL-6 levels—did not influence the outcomes, as demonstrated by the MANCOVA analysis (Supplement File S2), only the ANOVA results based on the 26 participants who completed the protocol are presented in this article. The intention-to-treat analysis, which included all 40 participants who initiated the study, is available in Supplement File S1.

### 3.3. Baseline Characteristics

The initial analysis, which included all participants enrolled at the beginning of the study, even those who did not complete all evaluation stages or the intervention period, indicated that the groups were homogeneous for all variables (Table 1). However, the analysis that included only the individuals who completed all stages of the study showed significant differences between groups for the inflammatory markers TNF-α and IL-6, as well as for muscle strength measures in the sit-to-stand and plantar flexion tests (Table 2).

### 3.4. Body Composition and Inflammatory Marker Outcomes

No significant differences were found between groups in body composition measures, vitamin D levels, or TNF-α and IL-6 at post-test (Table 3). However, a significant time × group interaction was observed for IL-6 (*p* = 0.03), indicating a trend toward a differential response, even though the post-test comparison between groups did not reach statistical significance. It should be noted that, although not significant, the control group showed an 11.9% increase in vitamin D levels, while the experimental group experienced a 2.2% decrease.

### 3.5. Muscle Strength and Functional Performance

Both groups showed significant improvements in five of the seven strength exercises, with the most pronounced gains in the lower limbs. The control group demonstrated significant improvements in all exercises, particularly in plantar flexion and leg press. In the functional tests, the control group exhibited greater variation in performance, although there were no statistically significant differences between groups at the end of the study.

It is noteworthy that the analysis performed using models adjusted for baseline values—accounting for the initial differences between groups in TNF-α and IL-6 levels, variables that could potentially influence the outcomes—showed no impact on the results presented (Supplement File S2).

## 4. Discussion

This study aimed to identify the effects of combining resistance training with vitamin D supplementation on variables related to body composition, muscle strength, and inflammatory status in older adults. Overall, supplementation at the dosage used in this research was not able to enhance the adaptations promoted by training in the outcomes analyzed, failing to confirm our initial hypothesis. Therefore, our study does not corroborate the findings of previous studies [6,22,23], which support the role of vitamin D in increasing muscle mass and strength, but it is closer to the results found in the meta-analysis by Antoniak et al. [13].

Regarding body composition, it is worth noting the scarcity of studies that have exclusively investigated the combination of resistance training with isolated vitamin D supplementation. In most cases, concomitant use of other supplements, such as calcium [24] and proteins [25], is observed. More recently, however, in a study with a sample similar to the present investigation, involving participants with vitamin D levels considered normal (above 30 ng/mL) [26], the combination of vitamin D supplementation alone with resistance training also did not result in significant effects on body composition.

Unlike the findings of this study, a clinical trial with older adults deficient in vitamin D (levels < 30 ng/mL) showed that supplementation with vitamin D (800 IU) and calcium (400 mg), followed by 10 weeks of resistance training, significantly reduced adipose tissue and body fat percentage [24]. The difference between these results and the findings of the present study may be related to the initially low levels of bioavailable vitamin D, which were corrected through supplementation during the study—something that did not occur in our experiment, which was conducted with a sample that had normal vitamin D levels throughout the entire intervention.

The effects found by Draxler et al. [24] may be explained by the inverse relationship between the increase in adipose tissue and the number of active vitamin D receptors, given that vitamin D plays an important role in adipocyte differentiation [27]. Another relevant factor is the role of vitamin D in regulating calcium homeostasis in various body tissues, including adipose tissue, particularly within the intracellular environment of adipocytes, as adequate calcium levels are associated with the induction of apoptosis in these cells [28].

In a similar study to the one described above [24] but conducted with an older population with normal vitamin D levels [29], as in our study, the results differed from those of Draxler et al. [24], aligning more closely with ours. Aschauer et al. [29], in a clinical trial combining resistance training (twice a week for 16 weeks) with vitamin D supplementation (800 IU) plus 400 mg of calcium, did not observe differences in body composition effects compared to the placebo group. These findings suggest that vitamin D appears to enhance the effects of resistance training only in individuals with vitamin D deficiency.

Studies using high doses of protein combined with vitamin D and other supplements have also shown relevant results. In a 12-week resistance training program, with supplementation starting six weeks earlier (30 g of protein, 500 IU of vitamin D, 400 mg of calcium, and 2.5 g of creatine), only the supplemented group showed initial improvements. During training, both groups improved lean mass and body composition, and by the end, no significant differences were found, suggesting that training alone compensated for the early gains from supplementation. Although our study used only vitamin D, the results are similar to those reported by Bell et al. [25], in which both groups achieved significant gains as a result of the training.

Contrary to the findings of Bell et al. [25] and our experiment, another study conducted with individuals aged 50 to 75 years who trained twice a week for 24 weeks [30] showed that the group receiving 40 g of protein and 2000 IU of vitamin D experienced significant increases in total lean mass and appendicular lean mass, as well as reductions in total body fat, while the control group showed no significant changes. Such contradictions can be explained by methodological differences between the studies, especially regarding the different supplementation dosages used.

From these observations, it appears that studies combining protein supplementation (30–40 g), even with lower doses of vitamin D (500–2000 IU), yielded better body composition outcomes than those without such supplementation, even when training periods were similar or longer. Thus, older adults’ responses to resistance training also seem to be enhanced when protein doses are combined with vitamin D supplementation.

This effect may be explained by the combined action of vitamin D and protein on protein balance, which is often negative in older adults, favoring muscle mass loss, a reduction in basal metabolic rate, and a possible increase in total body fat, but this was not tested in our experiment. The association of vitamin D with protein appears to act positively on protein synthesis in myotubes formed by the differentiation of satellite cells [22,23]. Myotubes, located in the cytoskeleton, have structural and intracellular transport functions, including proteins related to muscle contraction and myosin filament synthesis [31]. However, this mechanism has only been demonstrated in animal models [32], and the physiological process remains not fully elucidated in humans.

Regarding muscle strength, both groups in the present study showed significant improvements in almost all exercises between pre- and post-intervention, especially in the lower limbs. However, unexpectedly, significant differences were found between groups in some exercises (handgrip strength, plantar flexion, and leg press), with better results in the control group. Although both groups performed resistance training, which was expected to result in improvements, it was anticipated that the vitamin D-supplemented group would achieve greater gains, which was not confirmed. This difference may be attributed to selective dropout during the intervention, which resulted in a higher proportion of men in the control group. However, this explanation is not supported by previous studies, which have not found significant differences in strength gains between sexes, as demonstrated by Lemmer et al. [33]. Furthermore, no baseline differences were observed that could justify the control group’s superior performance in the tests mentioned. The only significant differences found between the groups at baseline were in the levels of TNF-α and IL-6, both inflammatory markers, with the control group showing lower values. However, the analysis taking this baseline difference into account did not identify any influence of this fact on the final results. Moreover, these differences disappeared by the end of the experiment, indicating not only the inefficacy of supplementation with 2000 IU/day of vitamin D regarding this outcome, but also that this does not represent a bias capable of influencing the better results observed in the control group [3]. Therefore, it is assumed that the larger number of participants who completed the study in the control group may have influenced this outcome.

In line with the findings of the present study, clinical trials using similar methodologies—although employing protein supplementation combined with vitamin D—also did not observe additional effects on muscle strength. Consistent with our results, Bell et al. [25] reported an increase in handgrip strength in both the experimental and control groups, with no significant differences between them. Similarly, another study combined resistance training with a drink containing 20 g of protein and 800 IU of vitamin D (or placebo) over six months, yielding comparable results [34]. Thus, contrary to what was initially proposed in our hypothesis, the available evidence, together with the findings of this study, suggests that vitamin D supplementation does not enhance the effects of resistance training on muscle strength in older adults.

Conversely, another study was conducted with 117 older adults with vitamin D deficiency [35]. The intervention lasted 12 months, with daily supplementation of 3 g calcium + 2000 IU vitamin D, or placebo. Strength training was performed three times a week on alternate days. The sample was divided into four groups: control; exercise + placebo; exercise + supplementation; and supplementation only. Muscle strength measurements were collected on the 3rd, 6th, 9th, and 12th month. Significant differences between the supplemented groups (with or without exercise) and the placebo groups were observed only in the third month. This result suggests that normalizing vitamin D levels was sufficient to promote muscle strength gains, without additional benefits from continued supplementation after this period.

When comparing the above studies, their experimental designs, and respective results, the differences observed can be explained by two physiological mechanisms associated with vitamin D levels. First, vitamin D binds to its nuclear receptor, altering mRNA transcription and promoting the synthesis of contractile proteins, which improves muscle strength. Second, vitamin D aids in the regulation of calcium (Ca^2+^), which is essential for muscle contraction through actin-myosin interaction [36,37].

Since the sample in the present study did not present vitamin D deficiency, it is possible that, as observed with body composition, the vitamin D dosage administered was not sufficient to produce additional effects in individuals with normal vitamin D levels. Alternatively, the hypothesis can be considered that no supplemental vitamin D dosage is effective for improving muscle strength in older adults who already have adequate levels of this nutrient.

Regarding inflammatory status, few studies have examined the relationship between resistance training combined with vitamin D supplementation and this outcome. Among them, one trial supplemented older adults for six weeks with a beverage containing 2.5 g creatine, 400 mg calcium, 500 IU vitamin D, and 1500 mg omega-3, twice daily, without training intervention [25]. At this stage, significant improvements were observed in the inflammatory markers investigated (TNF-α and IL-6). Supplementation was then maintained for another 12 weeks, now combined with resistance training. During this phase, TNF-α and IL-6 levels were further reduced. In contrast, another study already cited in the body composition analysis found significant changes only for TNF-α [30].

The results of the present research, unlike the above studies [25,30], did not indicate relevant effects of the interventions on TNF-α and IL-6 markers. However, participants already began the experiment with low values for these markers, which may have limited the intervention’s ability to reduce them further. Nevertheless, it is worth noting that the experimental group showed a slight decrease in IL-6 levels, whereas in the control group this marker increased. This finding may suggest a potential role of vitamin D in the downregulation of inflammatory markers, which can be explained by the presence of specific vitamin D receptors on macrophage membranes—cells involved in both tissue repair and inflammation modulation [38].

Finally, it is important to highlight that although vitamin D levels did not show significant differences in any of the analyses, a variation over time within the groups was observed (EG: 69.5 ± 23.5 vs. 68.0 ± 24.1; Δ%: –2.2; CG: 52.1 ± 15.2 vs. 58.3 ± 24.6), which may have influenced the results and represents a potential limitation of the study. Additionally, it is possible that the administered dosage was insufficient to promote relevant changes in the analyzed variables, especially considering that the participants did not present vitamin D deficiency. Another significant limitation was the sample loss associated with the pandemic context in which the study was conducted. The need to remove participants due to COVID-19 or other flu-like syndromes, combined with the inability to strictly control diet, negatively impacted the development and consistency of the study.

## 5. Conclusions

It is concluded that supplementation with 2000 IU/day of vitamin D in older adults with adequate levels of this vitamin was not effective in enhancing the adaptations induced by resistance training, performed twice a week over a 12-week period, in body composition, muscle strength, and markers of inflammatory status.

Despite the conclusions drawn, the limitations of the present study, particularly the small sample size, must be acknowledged. Given the relevance of this topic to older adults’ health, with potential implications for public health, the hypothesis that vitamin D may enhance the effects of resistance training on the analyzed variables cannot yet be ruled out. This is partly due to the limited number of existing studies and methodological differences in training and supplementation interventions. Therefore, further research is recommended to address these limitations and deepen the understanding of the interaction between these factors.

## Figures and Tables

**Table 1:** Subject Characteristics Mixed Models Analysis (n = 40).

Variable	EG (n = 22)	CG (n = 18)	*p*-Value	Mean Difference [IC95%]
Sex				
Female—n (%)	19 (86.4)	18 (77.7)		
Male—n (%)	3 (13.6)	4 (22.2)		
Age (years)	70.8 ± 4.8	70.1 ± 4.9	0.66	0.71 [−2.44; 3.86]
Body Composition				
Body mass (kg)	69.7 ± 12.5	69.4 ± 10.9	0.90	0.24 [−7.53; 8.00]
Height (m)	1.57 ± 0.10	1.59 ± 0.07	0.57	−0.15 [−0.68; 0.37]
BMI (kg/m^2^)	28.2 ± 3.8	27.6 ± 3.7	0.62	0.60 [−1.84; 3.04]
Lean mass (kg)	38.2 ± 7.4	39.6 ± 6.5	0.52	−1.33 [−5.46; 2.80]
Fat mass (kg)	29.4 ± 8.3	27.2 ± 6.6	0.38	2.21 [−2.82; 7.24]
Appendicular lean mass (kg)	16.7 ± 3.4	17.3 ± 3.7	0.60	−0.62 [−2.95; 1.70]
Body fat (%)	43.0 ± 7.8	40.5± 6.7	0.31	2.60 [−2.58; 7.77]
Blood Analysis				
Vitamin D (ng/mL)	66.8 ± 23.3	55.0 ± 18.6	0.14	11.80 [−3.93; 27.53]
TNF-α (pg/mL)	2.9 ± 11.7	0.0 ±0.0	0.27	2.92 [−2.47; 8.03]
IL-6 (pg/mL)	6.1 ± 9.5	2.4 ±1.4	0.13	3.71 [−1.18; 8.59]
Muscle Strength				
Sit-to-stand test (reps)	11.36± 4.1	10.8 ± 2.2	0.67	0.55 [−2.88; 3.98]
Handgrip strength (kg)	21.3 ± 7.9	25.3 ± 6.7	0.11	−4.01 [−8.87; 0.86]
Bench press—10 RM (kg)	10.0 ± 6.5	8.3 ± 5.1	0.38	1.72 [−2.12; 5.63]
Seated row—10 RM (kg)	11.9 ± 6.4	10.5 ± 4.1	0.43	1.46 [−2.24; 5.16]
Plantar flexion—10 RM (kg)	43.2 ± 21.7	34.4 ± 10.9	0.15	8.81 [−3.19; 20.81]
Leg press—10 RM (kg)	40.9 ± 18.7	33.1 ± 13.0	0.18	7.78 [−3.77; 19.34]
Leg extension—10 RM (kg)	15.5± 7.4	11.4 ± 7.2	0.07	4.05 [−0.28; 8.37]
Leg curl—10 RM (kg)	15.3 ± 6.9	13.4 ± 5.1	0.36	1.90 [−2.24; 6.05]
Pulldown—10 RM (kg)	16.8± 5.6	16.3 ± 6.7	0.78	0.57 [−3.56; 4.69]

EG: experimental group (resistance training + vitamin D); CG: control group (resistance training + placebo); BMI: body mass index; RM: repetition maximum; TNF-α: tumor necrosis factor-alpha; IL-6: interleukin-6; reps: repetitions; kg: kilogram; m: meter.

**Table 2:** Subject Characteristics (n = 26).

Variable	EG (n = 12)	CG (n = 14)	*p*-Value	Mean Differences [IC95%]
Sex				
Female—n (%)	11 (91.7)	11 (78.6)		
Male—n (%)	1 (8.3)	3 (21.4)		
Age (years)	70.6 ± 4.7	69.6 ± 4.6	0.82	0.94 [−2.85; 4.74]
Body Composition				
Body mass (kg)	68.7 ± 9.3	69.1 ± 9.2	0.80	1.60 [−6.72; 9.92]
Height (m)	1.55 ± 0.10	1.59 ± 0.10	0.40	−0.03 [−0.10; 00.04]
BMI (kg/m^2^)	28.4 ± 2.3	27.8 ± 3.8	0.19	1.05 [1.65; 3.75]
Lean mass (kg)	36.1 ± 3.9	39.5 ± 6.8	0.69	−1.92 [−7.35; 3.51]
Fat mass (kg)	30.4 ± 7.2	28.2 ± 5.7	0.49	2.67 [−2.53; 7.87]
Appendicular lean mass (kg)	15.9 ± 1.8	17.3 ± 3.9	0.41	−0.71 [−3.60; 2.17]
Body fat (%)	45.3 ± 5.2	41.5 ± 5.1	0.75	2.67 [−2.53; 7.87]
Blood Analysis				
Vitamin D (ng/mL)	69.5 ± 23.5	52.1 ± 15.2	0.49	17.36 [1.41; 33.32]
TNF-α (pg/mL)	4.2 ± 14.1	0.0 ±0.0	0.02	4.24 [−3.49; 11.98]
IL-6 (pg/mL)	6.4 ± 11.1	2.3 ±1.5	0.02	4.08 [−2.10; 10.26]
Muscle Strength				
Sit-to-stand test (reps)	13.3 ± 6.1	10.7 ± 2.3	0.04	2.45 [−1.06; 5.96]
Handgrip strength (kg)	19.8 ± 6.5	25.1 ± 7.3	0.46	−5.01 [−10.57; 0.54]
Bench press—10 RM (kg)	9.5 ± 3.5	8.0 ± 5.3	0.34	1.55 [−2.16; 5.25]
Seated row—10 RM (kg)	10.7 ± 5.0	10.2 ± 4.2	0.13	0.86 [−2.84; 4.57]
Plantar flexion—10 RM (kg)	43.6 ± 17.4	34.3 ± 11.6	0.09	11.55 [−0.67; 23.77]
Leg press—10 RM (kg)	38.1 ± 11.7	32.1 ± 13.7	0.94	8.69 [−2.700; 20.09]
Leg extension—10 RM (kg)	15.0 ± 4.5	11.6 ± 7.4	0.22	3.81 [−1.28; 8.90]
Leg curl—10 RM (kg)	13.6 ± 3.9	12.9 ± 5.0	0.20	1.31 [−2.550; 5.12]
Pulldown—10 RM (kg)	16.4 ± 4.5	16.1 ± 7.1	0.17	0.60 [−4.331; 5.50]

EG: experimental group (resistance training + vitamin D); CG: control group (resistance training + placebo); BMI: body mass index; RM: repetition maximum; TNF-α: tumor necrosis factor-alpha; IL-6: interleukin-6; rep: repetitions; kg: kilogram; m: meter.

**Table 3:** Results Across Groups and Time (n = 26).

Variable	EG Pre	EG Post	Δ%	*p*	Mean Change [IC95%]	CG Pre	CG Post	Δ%	*p*	Mean Change [IC95%]	*p*	Mean Differences [IC95%]
Body Composition												
Body mass (kg)	68.7 ± 9.3	67.9 ± 9.9	–1.1	0.36	0.75 [−0.91; 2.40]	69.1 ± 9.2	69.9 ± 9.8	1.1	0.26	−0.81 [−2.28; 0.654]	0.62	−2.00 [−10.22; 6.22]
BMI (kg/m^2^)	28.4 ± 2.3	28.1 ± 2.3	–1.1	0.14	0.35 [−0.12; 0.81]	27.8 ± 3.8	27.6 ± 3.5	–0.7	0.28	0.22 [−19; 0.64]	0.70	0.48 [−2.07; 3.03]
Lean mass (kg)	36.1 ± 3.9	35.8 ± 3.7	–0.8	0.52	0.23 [−0.50; 0.96]	39.5 ± 6.8	39.4 ± 6.7	–0.2	0.84	0.07 [−0.58; 0.71]	0.13	−3.07 [−1.06; 8.27]
Fat mass (kg)	30.4 ± 7.2	29.8 ± 7.6	–2.0	0.20	0.61 [−0.34; 1.57]	28.2 ± 5.7	28.0 ± 5.9	–0.7	0.68	0.17 [−0.67; 1.02]	0.51	1.81 [−3.78; 7.40]
Appendicular lean mass (kg)	15.9 ± 1.8	15.6 ± 1.9	–1.9	0.30	0.31 [−0.29; 0.90]	17.3 ± 3.9	17.2 ± 3.5	–0.6	0.64	0.12 [−0.41; 0.64]	0.19	−1.60 [−4.04; 0.84]
Body fat (%)	45.3 ± 5.2	44.9 ± 5.3	–0.9	0.84	0.45 [−4.08; 4.97]	41.5 ± 5.1	38.7 ± 11.6	–6.7	0.16	2.80 [−1.21; 6.81]	0.12	6.16 [−1.72; 14.04]
Blood Analysis												
Vitamin D (ng/mL)	69.5 ± 23.5	68.0 ± 24	–2.2	0.78	1.49 [−9.57; 12.54]	52.1 ± 15.2	58.3± 24.6	11.9	0.21	−6.17 [−15.97; 3.63]	0.33	9.71 [−10.65; 30.06]
TNF-α (pg/mL)	4.2 ± 14.1	3.5 ± 9.0	–16.7	0.56	0.72 [−1.76; 3.19]	0.0 ± 0.0	0.0 ± 0.0	0.0	0.0	0.0 [0.0; 0.0]	0.16	3.53 [−1.44; 8.50]
IL-6 (pg/mL)	6.4 ± 11.1	3.6 ± 6.1	–43.8	0.91	2.75 [−0.48; 5.99]	2.3 ± 1.5	4.2 ± 5.5	82.6	0.18	−1.92 [−4.78; 0.95]	0.80	−0.59 [−5.43; 4.24]
Muscle Strength												
Sit-to-stand (reps)	13.3 ± 6.1	14.2 ± 4.1	6.7	0.15	−0.91 [−2.17; 0.35]	10.7 ± 2.3	13.2 ± 2.6	23.4	0.00	−2.57 [−3.68; 1.45]	0.52	0.90 [1.94; 3.74]
Handgrip strength (kg)	19.8 ± 6.5	19.8 ± 6.3	0.0	0.96	0.05 [−1.91; 2.01]	25.1 ± 7.3	26.9 ± 8.0	7.2	0.05	−1.75 [−3.49; −0.01]	0.02	−7.11 [−13.22; −1.00]
Bench press—10 RM (kg)	9.5 ± 3.5	12.5 ± 6.9	31.6	0.26	−2.96 [−8.21; 2.30]	8.0 ± 5.3	20.1 ± 9.6	151.3	0.00	−12.14 [−16.80; −7.48]	0.04	−7.68 [−14.83; −0.53]
Seated row—10 RM (kg)	10.7 ± 5.0	17.3 ± 5.4	61.7	0.02	−6.59 [−11.91; −1.27]	10.2 ± 4.2	22.5 ± 9.8	120.6	0.00	−12.32 [−17.04; −7.61]	0.16	−5.23 [−12.05; 1.60]
Plantar flexion—10 RM (kg)	43.6 ± 17.4	70.9 ± 19.2	62.6	0.00	−27.27 [−42.25; −12.30]	34.3 ± 11.6	96.4 ± 25.6	296.7	0.00	−62.14 [−75.42; −48.87]	0.01	−25.52 [−44.72; -.31]
Leg press—10 RM (kg)	38.1 ± 11.7	61.8 ± 19.4	62.2	0.00	−23.64 [−38.35; −8.92]	32.1 ± 13.7	87.1 ± 25.8	171.3	0.00	−55.00 68.05; −41.96]	0.01	−25.33 [−44.72; −5.93]
Leg extension—10 RM (kg)	15.0 ± 4.5	23.2 ± 5.5	54.7	0.12	−8.18 [−14.34; −2.02]	11.6 ± 7.4	27.5 ± 12.1	137.1	0.00	−15.89 [−21.38; −10.43]	0.29	−4.32 [−12.50; 3.86]
Leg curl—10 RM (kg)	13.6 ± 3.9	27.5 ± 18.4	102.2	0.00	−13.86 [−22.33; −5.40]	12.9± 5.0	25.5± 8.0	97.7	0.00	−12.68 [−20.18; −5.18]	0.72	1.96 [−9.34; 13.26]
Pulldown—10 RM (kg)	16.4 ± 4.5	21.4 ± 4.5	30.5	0.06	−5.00 [−10.22; 0.22]	16.1± 7.1	25.0± 9.8	55.2	0.00	−8.93 [−13.55; −4.30]	0.29	−3.64 [−10.27; 2.99]

EG: experimental group (resistance training + vitamin D); CG: control group (resistance training + placebo); Pre: pre-intervention; Post: post-intervention; Δ%: percent change; IC95%: 95% confidence interval for difference; BMI: body mass index; RM: repetition maximum; rep: repetition; kg: kilogram; %: percent; TNF-α: tumor necrosis factor-alpha; IL-6: interleukin-6.

## Data Availability

The data presented in this study are available on request from the corresponding author. Due to the inclusion of human subjects, public access to the data is restricted, as no authorization was obtained from the participants to share their information openly.

## Supplementary Materials

The following supporting information can be downloaded at https://www.mdpi.com/article/10.3390/ijerph22111695/s1: File S1: title Results Across Groups and Time Mixed Model (n = 40); File S2: title MANCOVA Results Adjusted for Baseline Covariates.

## Abbreviations

The following abbreviations are used in this manuscript:

1,25(OH)_2_D	1,25-dihydroxyvitamin D
25(OH)D	25-hydroxyvitamin D
7-DHC	7-dehydrocholesterol
ALM	Appendicular Lean Mass
BMI	Body Mass Index
Ca^2+^	Calcium ion
CG	Control Group
CNPJ	Brazilian National Registry of Legal Entities
DEXA	Dual-Energy X-ray Absorptiometry
EG	Experimental Group
ELISA	Enzyme-Linked Immunosorbent Assay
FITT	Frequency, intensity, time and type of exercise
HUPE	Pedro Ernesto University Hospital
IL-6	Interleukin-6
Kg	Kilogram
LABSAU	Physical Activity and Health Promotion Laboratory
M	Meter
mRNA	Messenger Ribonucleic Acid
PTH	Parathyroid Hormone
Rep	Repetitions
RM	Repetition Maximum
SPSS	Statistical Package for the Social Sciences
TNF-α	Tumor Necrosis Factor-alpha
UERJ	State University of Rio de Janeiro
%	Percent

## Appendix A

The CONSORT2025 checklist

**Section**	**Item**	**Description/Comment**
1. Title and Abstract	Identification as RCT	✅ Yes—stated in the title and abstract.
1. Title and Abstract	Structured summary	✅ Includes design, participants, interventions, outcomes, and results.
2. Introduction	Background and rationale	✅ Clearly described in the text.
2. Introduction	Objectives/hypotheses	✅ Defined: to investigate the effects of training + vitamin D.
3. Methods	Study design	✅ RCT, double-blind, controlled.
3. Methods	Setting and dates	✅ LABSAU/UERJ and HUPE, January–December 2022.
3. Methods	Ethics and registration	✅ HUPE Ethics Committee, Opinion No. 3.655.940.
3. Methods	Inclusion/exclusion criteria	✅ Detailed presentation.
3. Methods	Recruitment	✅ At LABSAU/UERJ; assessments at HUPE.
3. Methods	Intervention details	✅ Training and supplementation protocols described.
3. Methods	Control group	✅ Identical-appearing placebo.
3. Methods	Primary outcomes	✅ Body composition, muscle strength, inflammatory markers.
3. Methods	Secondary outcomes	✅ None.
3. Methods	Sample size calculation	✅ G*Power, α = 0.05; β = 0.15; effect size from literature.
3. Methods	Random sequence generation	✅ Simple randomization with sex and age stratification.
3. Methods	Allocation concealment	✅ Performed by an independent researcher.
3. Methods	Blinding	✅ Participants and assessors blinded to supplement allocation.
3. Methods	Statistical analysis	✅ Two-factor ANOVA, Bonferroni, Shapiro–Wilk, *p* ≤ 0.05.
3. Methods	Per-protocol analysis	✅ Included only participants who met adherence criteria.
4. Results	Participant flow	✅ Flowchart: 40 randomized, 26 analyzed.
4. Results	Baseline characteristics	✅ Values and *p*-values showing group homogeneity.
4. Results	Outcome data	✅ Pre/post/Δ% values with significance reported.
4. Results	Adverse events	✅ None reported. Method of collection needs description.
5. Discussion	Interpretation and limitations	✅ Included in the manuscript.
6. Other	Funding/conflicts of interest	✅ Include before references, if applicable.
✅ Confirmation that the item has been completed.		
