# Supplementary material for: Effects of Resistance Training Combined with Vitamin D Supplementation on Health-Related Variables in the Elderly: Muscle Strength, Body Composition, and Inflammatory Status
Source: Int J Environ Res Public Health. 2025 Nov 10;22(11):1695. doi: 10.3390/ijerph22111695 (PMC12652358; doi:10.3390/ijerph22111695)
Supplement: Supplementary file 1 [file ijerph-22-01695-s001.zip › ijerph-3910875-supplementary.pdf]

SUPPLEMENT 1

Table X. Results Across Groups and Time Mixed Model (n = 40)

Variable	EG Pre	EG Post	Δ%	p	Mean Change [IC95%]	CG Pre	CG Post	Δ%	p	Mean Change [IC95%]	p (Group Difference)	Mean Difference [IC95%]
**Body Composition**												
Body mass (kg)	69.7 ± 12.5	71.0 ± 12.1	-1.2	0.85	-0.43[-4.89; 4.04]	69.4 ± 10.9	69.2 ± 9.8	1.2	1.0	0.22[0.22; -0.22]	0.82	0.89[-6.98; 8.75]
BMI (kg/m²)	28.2 ± 3.8	28.3 ± 3.2	-1.1	0.95	0.05[-1.33; 1.42]	27.6 ± 3.7	27.5± 3.7	-0.7	1.0	0.12[0.12; 0.12]	0.58	0.68[-1.77; 3.12]
Lean mass (kg)	38.2 ± 7.4	39.3 ± 7.9	-0.8	0.98	-0.21[-1.49; 1.45]	39.6 ± 6.5	39.5 ± 6.2	-0.3	1.0	0.06[0.06; -0.06]	0.55	-1.26[-5.45; 2.94]
Fat mass (kg)	29.4 ± 8.3	29.3 ± 7.9	-2.0	0.89	0.19[-2.58; 2.95]	27.2 ± 6.6	27.2 ± 6.8	-0.7	1.0	-0.04[-0.04; -0.04]	0.44	1.98[-3.15; 7.12]
Appendicular lean mass (kg)	16.7 ± 3.4	17.2 ± 3.5	-1.9	0.94	-0.05[-1.40; 1.30]	17.3 ± 3.7	17.2 ± 3.3	-0.6	1.0	0.13[0.13; 0.13]	0.71	-0.45[-2.81; 1.94]
Body fat (%)	43.0 ± 7.8	42.5 ± 7.1	-0.9	0.90	0.22[-3.12; 3.56]	40.5± 6.7	38.1 ± 11.7	-6.7	0.18	-2.33[-1.15; 5.80]	0.13	4.70[-1.40; 10.79]
**Blood Analysis**												
Vitamin D (ng/mL)	66.8 ± 23.3	70.1 ± 27.5	-2.2	0.58	-3.24[-15.09; 8.62]	55.0 ± 18.6	60.4 ± 25.1	11.9	0.37	-5.32[-17.18; 6.54]	0.31	9.71[-9.54; 28.98]

TNF-α (pg/mL)	2.9 ± 11.7	2.4 ± 7.6	-16.7	0.77	0.49[-3.25; 4.23]	0.0 ±0.0	0.0 ± 0.0		0.0	0.0[0.0; 0.0]	0.29	2.43[-2.20; 7.05]
IL-6 (pg/mL)	6.1 ± 9.5	4.1± 6.1	-43.8	0.19	2.01[-1.08; 5.11]	2.4 ±1.4	3.8 ± 5.3	82.6	0.35	-1.44[-4.54; 1.65]	0.90	0.25[-3.88; 4.39]
**Muscle Strength**												
Sit-to-stand (reps)	11.4± 4.1	13.4 ± 4.6	6.8	0.13	-2.07[-5.91; 1.77]	10.8 ± 2.2	12.9 ± 2.6	23.4	0.15	-2.13[-7.39; 3.14]	0.70	0.50[-2.03; 3.02]
Handgrip strength (kg)	21.3 ± 7.9	22.7 ± 7.0	0.0	0.78	-0.34	25.3 ± 6.7	26.7 ± 7.5	7.2	1.0	-1.44[-1.44; -1.44]	0.04+	-5.10[-10.04; -0.16]
Bench press – 10 RM (kg)	10.0 ± 6.5	14.8 ± 9.7	31.6	0.00	-4.77[-7.97; 1.57]	8.3 ± 5.1	19.8 ± 9.1	151.3	0.00	-11.56[-15.31; -7.81]	0.09	-5.07[-10.47; 0.59]
Seated row – 10 RM (kg)	11.9 ± 6.4	18.6± 8.0	61.7	0.00	-6.68[-9.88; -3.48]	10.5 ± 4.1	22.5 ± 9.1	120.6	0.00	-12.03[-15.79; -8.28]	0.16	-3.89[-9.31; 1.54]
Plantar flexion – 10 RM (kg)	43.2 ± 21.7	71.4 ± 24.6	62.6	0.00	-28.18[-38.14; -18.22]	34.4 ± 10.9	96.5± 24.1	181.0	0.00	-61.25[-72.92; -49.57]	0.01+	-24.26[-40.53; -7.99]
Leg press – 10 RM (kg)	40.9 ± 18.7	65.0 ± 25.21	62.2	0.00	-24.10[-33.24; -14.94]	33.1 ± 13.0	85.6± 24.5	171.3	0.00	-52.50[-63.23; -41.23]	0.01+	-20.63[-35.41; -5.84]
Leg extension – 10 RM (kg)	15.5± 7.4	37.0 ± 64.94	54.7	0.03	-21.59[-40.37; -2.81]	11.4 ± 7.2	26.8± 11.5	137.1	0.17	-15.47[-37.49; 6.55]	0.48	10.17[-18.42; 38.76]
Leg curl – 10 RM (kg)	15.3 ± 6.9	25.8 ± 14.9	102.2	0.00	-10.46[-15.53; -5.38]	13.4 ± 5.1	25.3± 7.6	97.7	0.00	-11.88[-17.82; -5.92]	0.91	0.48[-7.77; 8.74]

Pulldown – 10 RM (kg)	16.8± 5.6	22.5 ± 7.3	30.5	0.00	-5.68[-8.80; -2.56]	16.3 ± 6.7	25.3 ± 9.2	55.3	0.00	-9.06[-12.72; -5.41]	0.29	-2.81[-8.14; 2.51]

EG: experimental group (resistance training + vitamin D); CG: control group (resistance training + placebo); Pre: pre-intervention; Post: post-intervention; Δ%: percent change; IC95%: 95% confidence interval for difference; BMI: body mass index; RM: repetition maximum; rep: repetition; kg: kilogram; %: percent; TNF-α: tumor necrosis factor-alpha; IL-6: interleukin-6.

SUPPLEMENT 2

Table X. MANCOVA Results Adjusted for Baseline Covariates

Source of variation	Multivariate test	Value	F	gl_1	gl_2
Grupo	Pillai's Trace	0.559	1.829[b]	9.000	13.000
TNF-α	Pillai's Trace	0.668	2.908[b]	9.000	13.000
IL-6	Pillai's Trace	0.681	3.084[b]	9.000	13.000

Table Y. Group Differences Post Intervention - Adjusted for Covariates

Variables	EG (n=12)		CG (n=14)		p	Mean Difference [IC95%]
	Mean	IC95%	Mean	IC95%		
Sit-to-stand (reps)	14.420[a]	[12.337; 16.503]	13.099[a]	[11.262; 14.936]	0.34	-1.321 [-1.515; 4.157]
Handgrip strength (kg)	19.457[a]	[14.669; 24.244]	27.213[a]	[22.991; 31.434]	0.02	-7.756 [-14.273; -1.238]
Bench press – 10 RM (kg)	12.016[a]	[6.355; 17.677]	20.559[a]	[15.567; 25.551]	0.03	-8.543 [-16.250; -0.836]
Seated row – 10 RM (kg)	17.269[a]	[11.797; 22.742]	22.503[a]	17.677; 27.329]	0.16	-5.234 [-12.684; 2.217]
Plantar flexion – 10 RM (kg)	71.696[a]	[56.559; 86.833]	95.810[a]	[82.462; 109.158]	0.02	-24.115 [-44.723; -3.506]
Leg press – 10 RM (kg)	62.639[a]	[47.096; 78.183]	86.498[a]	[72.791; 100.204]	0.03	-23.858 [-45.019; -2.697]
Leg extension – 10 RM (kg)	22.561[a]	[16.088; 29.034]	27.988[a]	[22.280; 33.696]	0.21	-5.427 [-14.240; 3.386]
Leg curl – 10 RM (kg)	27.781[a]	[18.779; 36.784]	25.315[a]	[17.376; 33.253]	0.68	2.466 [-9.790; 14.722]
Pulldown – 10 RM (kg)	21.464[a]	[16.239; 26.688]	24.922[a]	[20.314; 29.529]	0.32	-3.458 [-10.571; 3.655]
